# Getting to zero: Impact of a device to reduce blood culture contamination and false-positive central-line–associated bloodstream infections

**DOI:** 10.1017/ice.2022.284

**Published:** 2023-09

**Authors:** Lucy S. Tompkins, Vivian Tien, Alexandra N. Madison

**Affiliations:** 1 Division of Infectious Diseases and Geographic Medicine, Department of Medicine, Stanford University School of Medicine, Stanford, California; 2 Department of Infection Prevention and Control, Stanford Health Care, Stanford, California

## Abstract

**Objective:**

To assess the impact of initial specimen diversion device (ISDD) on inpatient and emergency department blood culture contamination (BCC), central-line–associated bloodstream infection (CLABSI) standardized infection ratios (SIRs), and antibiotic administration.

**Design:**

Single-center quasi-experimental prospective cohort study wherein phlebotomists used traditional venipuncture with or without the ISDD while registered nurses (RNs) used traditional venipuncture.

**Method:**

BCC events among phlebotomists and RNs were observed and compared from March 17, 2019, through January 21, 2020, defined by contaminant detection in 1 of 4 bottles for matched sets or 1 of 2 bottles in both subsets for coagulase negative staphylococci. CLABSIs throughout this period were recorded and SIRs were calculated. Enhanced oversight took place through July 21, 2019, with chart review assessing antibiotic use for patients with possible BCC.

**Results:**

Overall, 24% of blood cultures obtained were from patients in intensive care. Phlebotomists using traditional venipuncture (n = 4,759) had a 2.3% BCC rate; phlebotomists using the ISDD (n = 11,202) had a 0% BCC rate. RNs drew 7,411 BCs with a 0.8% BCC rate. The CLABSI SIR was decreased from 1.103 in 2017 and 0.658 in 2018 to 0.439 in 2019. The CLABSI incidence was 33%–64% of predicted value for each 2019 quarter. This range fell to 18%–37% after the exclusion of likely false-positive results. Among 42 patients with possible BCC under enhanced oversight, 2 patients were treated with prolonged antibiotic courses.

**Conclusions:**

ISDD use by phlebotomists was associated with BCC reduction and reduced false-positive CLABSI results. This patient-care quality improvement could constitute sustainable antibiotic stewardship expansion.

Blood cultures are the most important test used to diagnose sepsis. Approximately 8% of all blood cultures are positive, but published studies suggest that 20%–60% of positive cultures are false positives resulting from skin contaminants.^1–3^ Blood culture contamination (BCC) events may cause harm through misdiagnoses, inappropriate antimicrobial therapies, longer lengths of stay with attendant healthcare-associated infection risks and economic burdens.^1–9^ Moreover, false-positive central-line–associated bloodstream infections (CLABSIs) raise the standardized infection ratio (SIR) as defined by the National Healthcare Safety Network (NHSN).

Blood cultures may become contaminated when venipuncture dislodges squamous epithelial cells harboring superficial microorganisms or subepithelial bacteria and the ensuing flow of blood ferries this organic debris to the culture medium.^10^ In our experience, patients who have poor venous access are those most likely to have BCCs. The percentage of all positive blood cultures that yield contaminants depends upon the technique of venipuncture, antisepsis, glove use, patient body draw site, skin color and habitus, sample volume, transport time, blood culture kit, in-service training, and operator technique review, among other factors.^1–3,11^

A study on febrile patients presenting to an emergency department (ED) demonstrated that the use of an initial specimen diversion device (ISDD), which sequesters 1.5–2.0 mL blood and any accompanying skin contaminants into a separate chamber before opening an independent sterile blood flow path to a culture bottle, resulted in a significant decrease in BCC events compared with traditional venipuncture.^12^ To our knowledge, no data have been published on the impact of ISDD use on inpatient BCC and CLABSI reporting.

## Methods

We assessed the impact of ISDD utilization versus traditional venipuncture on BCC among adult inpatients and ED patients, and on the CLABSI SIR, during the study period. Whether inpatients with possibly contaminated blood cultures received prolonged courses of antibiotics was determined by chart review.

### Setting and study design

We conducted a quasi-experimental prospective cohort study at Stanford University Hospital, a 610-bed, urban, referral, academic medical center serving adults. We compared BCC rates among phlebotomists using either an ISDD (Steripath Gen2, Magnolia Medical Technologies, Seattle, WA) or traditional venipuncture while registered nurses (RNs) continued using traditional venipuncture. The study was carried out with enhanced oversight from March 17, 2019, to July 21, 2019, under the direction of the hospital epidemiologist with direct supervision by the Stanford Health Care (SHC) laboratory services phlebotomy supervisor. Thereafter, phlebotomy team members continued to use the ISDD without supervision or feedback until January 21, 2020. Under enhanced oversight, the charts of 42 patients who had contaminated cultures were reviewed by 2 authors (V.T. and L.S.T., not blinded) to assess clinical reasoning behind antibiotic treatment among those who received antimicrobials. The number of CLABSIs attributed to possibly contaminated blood cultures throughout the full study was obtained through the laboratory information system.

### Laboratory methods and definitions

The SHC laboratory uses BD BACTEC Plus, Aerobic/F culture vials, and BD BACTEC Plus Anaerobic/F culture vials (Becton Dickinson, Franklin Lakes, NJ). A matched blood culture set consists of 2 two-bottle subsets (4 bottles total with each requiring a 10-mL blood sample), where subset 1 contains 2 aerobic bottles and subset 2 contains 1 aerobic and 1 anaerobic bottle (3 aerobic bottles, 1 anaerobic bottle). Subsets for each matched set were drawn from separate sites through peripheral venipuncture. No line draws were included in the analysis. Following Clinical and Laboratory Standards Institute (CLSI) guidelines, the organisms the laboratory considers potential skin contaminants include coagulase-negative staphylococci (CoNS), viridans streptococci, *Corynebacterium* spp, *Cutibacterium acnes*, *Bacillus* spp, and *Micrococcus* spp.^13^ The microbiology laboratory reports the results for each bottle, and if only 1 of 4 bottles is positive with a skin organism as listed above, this was recorded as a contaminated matched set. We also recorded the number of matched sets having 1 of 4 bottles positive for vancomycin-resistant enterococci (VRE) or *Candida* spp. However, according to NHSN definitions, a single bottle of VRE or *Candida* spp must be reported by the infection preventionist as a CLABSI for patients with central venous access lines.^14^ We defined the BCC rate as the proportion of contaminated matched sets among observed matched sets for any given group.

### Patients and phlebotomists

Prior to the study, staff from Magnolia Medical Technologies trained members of the phlebotomy team (∼120) to properly use the ISDD. During the period of enhanced oversight from March 17, 2019, to July 21, 2019, phlebotomists were strongly encouraged to use the ISDD for all patients, including ‘hard stick’ patients with difficult venous access whenever possible. After July 21, 2019, there was no active supervision or encouragement of phlebotomists to use the ISDD.

### Blood culture collection

Phlebotomy team members routinely wore sterile gloves and a procedure mask when disinfecting the venipuncture site with chlorhexidine sponges. Phlebotomists disinfected blood-culture bottle tops with alcohol wipes prior to inoculation. RNs performing venipuncture used these same traditional methods of skin and blood-culture bottle-top preparation with appropriate personal protective equipment. Laboratory staff labeled and recorded blood cultures drawn using the ISDD.

### Data collection

We compared the proportion of contaminated blood cultures drawn by phlebotomists using traditional venipuncture with the proportion of contaminated blood cultures drawn using the ISDD from March 17, 2019, to January 21, 2020. We compared the total number of CLABSIs and standardized infection ratios (SIRs) reported to the NHSN for years 2017, 2018, and 2019.

### Statistical analysis

The significance of phlebotomy outcomes (ISDD vs traditional venipuncture) was determined using the Fisher exact test with *P* < .01 considered significant.

### Ethics

This study was conducted as part of a hospital-sponsored quality improvement project and Stanford University School of Medicine Institutional Review Board approval was waived.

## Results

### Sample collection and ISDD impact on BCC

Approximately 24% of all blood cultures were collected from patients in intensive care. Throughout the study, phlebotomists obtained 91% of the blood-culture sets from inpatients and 45% of the blood-culture sets from patients presenting to the emergency department (ED), and RNs drew the remaining 9% and 55% of blood cultures from inpatients and ED patients, respectively. Overall, phlebotomists used the ISDD to draw 11,202 matched blood culture sets, with a single matched set considered possible contamination (only 1 of 4 bottles was positive with VRE) (Table 1). In comparison, phlebotomists using traditional venipuncture to draw 4,759 sets had 111 contaminated matched sets and a 2.3% BCC rate (Fisher exact *P* < .001). Phlebotomists used the ISDD for an average of 70% of their blood draws over the entire study period (81% during enhanced oversight versus 60% while unsupervised). RNs drew a total of 7,411 matched sets with a BCC rate of 0.8%. The overall BCC rate was 0.73% (172 of 23,372).

**Table 1.** Blood Culture Collection and Contamination Event Distribution as Collected by Registered Nurses (RNs) and Phlebotomists Using Either Traditional Venipuncture or the Initial Specimen Diversion Device, March 17, 2019–January 21, 2020

Collection Method	Matched Sets, No.^a^	Contamination Events, No.	%
RN (traditional)	7,411	60	0.8
Phlebotomy (traditional)	4,759	111	2.3
Phlebotomy (ISDD)	11,202	1	0.0
Phlebotomy (all)	15,961	112	0.7
RN + phlebotomy (all)	23,372	172	0.7

Note. ISDD, initial specimen diversion device; RN, registered nurse.

a Matched sets include 2 sets of blood cultures, 4 bottles in total, drawn within a 24-hour period.

### Impact on CLABSIs

The total number of CLABSIs reported in the 2 previous years declined from 68 in 2017 and 48 in 2018 to 31 in 2019 and the SIR declined from 1.103 in 2017 and 0.658 in 2018 to 0.439 in 2019 (Table 2). During the 10-month study, a single possible contaminant-associated CLABSI (only 1 of 4 bottles was positive with VRE) was recorded with ISDD use. Differing significantly from the ISDD group, 12 contaminant-associated CLABSIs were observed when phlebotomists used traditional venipuncture (Fisher exact *P* < .001). Observed contaminant species included VRE, *Enterococcus faecalis*, *Candida* spp, and CoNS.

**Table 2.** Central-Line–Associated Bloodstream Infection (CLABSI) Standardized Infection Ratios (SIRs) and CLABSI Reports to the National Healthcare Safety Network (NHSN) Among Phlebotomists Using Traditional Blood Culture Methods

Year	SIR	CLABSI, No.	
		NHSN-Reported	Contaminant-Associated^a^
2017	1.103	68	17
2018	0.658	45	17
2019	0.439	31	13

a Includes reports in which 1 of 4 bottles were positive for vancomycin-resistant enterococci or *Candida* spp, or reports in which 1 of 2 bottles in both subsets were positive for coagulase-negative staphylococci within a 24-h period.

**Fig. 1.**
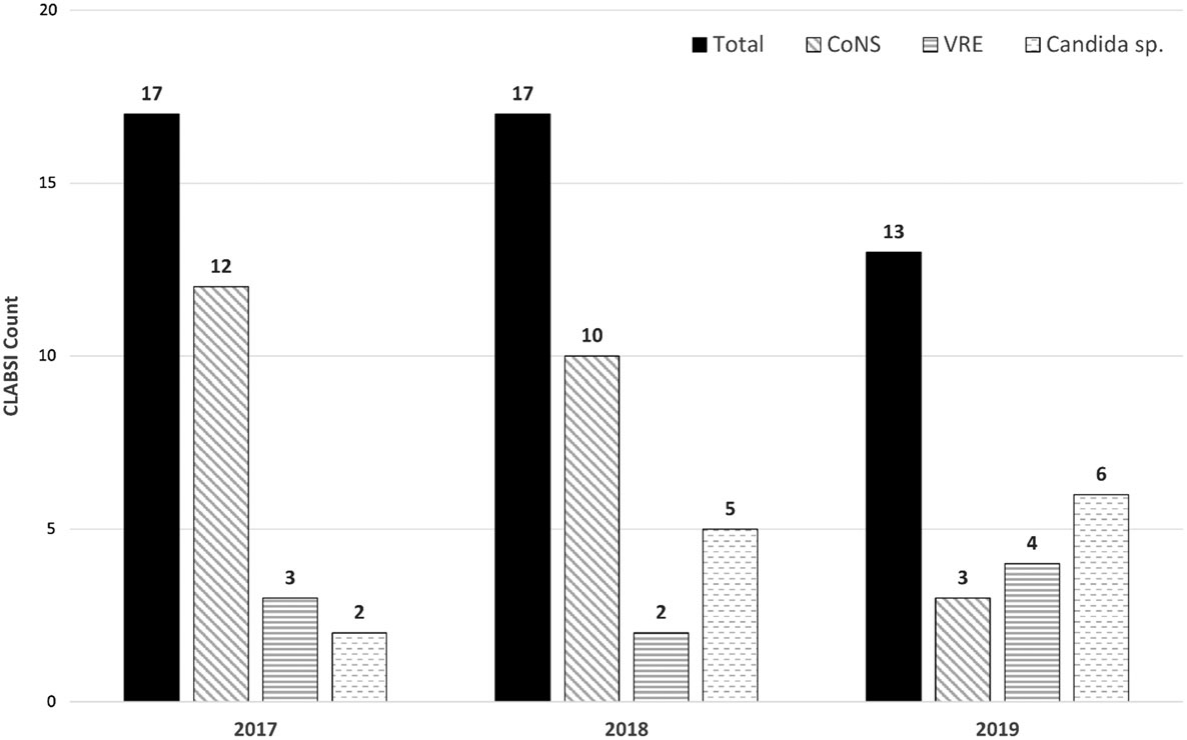
Annual central-line–associated bloodstream infection (CLABSI) contaminant strain distribution [predominantly coagulase-negative staphylococci (CoNS), vancomycin-resistant enterococci (VRE) and *Candida* spp] observed while using traditional methods to collect blood cultures in 2017, 2018, and 2019.

The impact of contaminant-associated CLABSIs on the SIR as determined by the NHSN statistics calculator was substantial.^15^ Exclusion of the probable contaminant-associated CLABSIs each quarter of 2019 resulted in a marked reduction in the SIR, ranging from 33% to 57% (Table 3). The observed increase in the SIR in quarter 4 (Q4) should be considered in the context of operational challenges for the phlebotomy team related to the opening of a new 360-bed hospital. This increase may have been due to logistical problems a reduced number of patients had blood cultures drawn with the ISDD during this period. The distribution of contaminant species from 2017 to 2019 was stable (Fig. 1).

**Table 3.** Quarterly Standardized Infection Ratio (SIR) Distribution for Central-Line–Associated Bloodstream Infection (CLABSI) Observations With and Without Likely Contaminants Included

Quarter	Predicted CLABSIs	Likely Contaminants Included		Likely Contaminants Excluded		
		Observed CLABSIs, No.	SIR	Observed CLABSIs, No.	SIR	SIR Reduction, %
2019 Q1	17.1	7	0.41	3	0.18	57
2019 Q2	16.6	6	0.36	4	0.24	33
2019 Q3	18.0	6	0.33	4	0.22	33
2019 Q4	18.8	12	0.64	7	0.37	42

### Impact on antibiotic utilization

During the initial study period, 2 authors (infectious diseases physicians) performed a retrospective chart review of 42 patients (44 episodes) with possible BBCs collected. Of the 48 blood-culture–matched sets in question, 90% contained CoNS, 4% contained viridans streptococci, 6% contained *Micrococcus* spp, and 4% contained *Bacillus* spp. Of the 42 patients with blood cultures positive for CoNS (1 of 2 bottles in both sets), 2 were prescribed vancomycin for >48 hours (4 and 7 days, respectively). Although the consulting infectious diseases physicians thought these were likely contaminants because subsequent blood cultures were negative, they recommended completing a short course of antibiotics to “cover” a possible line infection.

## Discussion

This study showed that blood-culture contamination was dramatically reduced when phlebotomists used the ISDD on both inpatients and ED patients. Conversely, according to definitions outlined herein, we observed 172 patients with contaminated blood cultures drawn via traditional venipuncture, who could have suffered potential harm, including 2 patients who may have received excess antibiotics.^16^

The overall reduction in BCC was accompanied by a reduction in reported CLABSIs in 2019 compared with 2017 and 2018. Although the reduction in CLABSIs between 2017 and 2018 may have been associated with the implementation of a more active skin preparation via swabbing with a larger volume of chlorhexidine, we consider the intervention described here to be the sole contributor to the subsequent decreased CLABSI SIR observed throughout 2019. False-positive CLABSIs may not only compromise clinical care but also have significant implications for benchmark rankings. During the year of the study, our medical center achieved a top-10 ranking in patient safety from Vizient, a consortium of 101 academic medical centers that ranks each member on healthcare-associated infection rates and many other factors. This achievement is due, in part, to our low CLABSI incidence.

We also noted a decrease in number of blood-culture sets containing a single bottle of either *Candida* spp or VRE. In the authors’ experience, enterococci and other gram-positive cocci replicate to very high numbers if present in an intravascular focus (eg, central line) and are usually present in all 4 bottles. By NHSN surveillance definitions, a single bottle of a matched 4-bottle set containing VRE is considered to be a true CLABSI, despite published studies showing that VRE can be present on the skin of patients who carry the organism in the gastrointestinal tract.^17,18^ At present, a single blood-culture bottle containing *Candida* spp is also considered evidence of true candidemia based on a historical clinical evaluation.^19^ However, at that time, laboratory guidelines recommended that matched blood-culture sets should consist of 2 aerobic bottles and 2 anaerobic bottles. Our laboratory uses 3 aerobic bottles in each 4-bottle set, and in our experience, at least 2 aerobic bottles are likely to be positive in patients with candidemia. As this pertains to the definition of BCC used here, we invite further input and future studies on this topic.

The study had several strengths. To the best of our knowledge, this study provides data from the largest number of blood cultures obtained using the ISDD, and it is the only published study using the ISDD to obtain blood cultures on both inpatients (including those in intensive care) and ED patients. The study also demonstrated that use of the ISDD markedly reduced the number of BCC events to a greater extent than has been previously observed.^20–23^ Moreover, ours is the first study to address the impact of contamination on false-positive CLABSIs. Notably, phlebotomists performing traditional venipuncture without the ISDD likely had higher BCC rates than RNs because phlebotomists drew most blood samples from inpatients, including those in the intensive care units and those considered to be ‘hard stick’ patients, whereas RNs drew blood samples primarily from ED patients who overall are not as difficult to draw from. Operator bias was reduced because the same phlebotomists used both the ISDD and traditional venipuncture throughout the study and particularly with the opening of the new hospital (a significant walk from the old hospital), which was associated with a decrease in usage of the ISDD by phlebotomists serving both hospitals.

This study had several limitations. It was restricted to a single center. Phlebotomists only used the ISDD with ∼70% of eligible patients overall, and we were not able to determine the reasons for decisions behind the choice of method. For practical reasons, we did not randomize members of the phlebotomy team to use traditional venipuncture versus the ISDD nor did we randomize the patients. Moreover, we did not control for phlebotomist technique by having 1 blood-culture set drawn via traditional venipuncture and the other drawn via the ISDD. Finally, it was not possible to delineate the data by patient and staff details beyond what has been presented here.

These results demonstrate that it is possible to eliminate BCC and thus “get to zero” when the ISDD is successfully employed, in line with the recently updated CLSI recommendation to reduce BCC below a 1% rate.^24^ By reducing BCC, this novel technology can have positive effects on the quality of patient care by facilitating correct diagnoses, reducing false-positive CLABSI reporting, and sustainably improving antibiotic stewardship practices with minimal training and oversight.
